# COVID-19 Breakthrough Infection Among Vaccinated Population in the United Arab Emirates

**DOI:** 10.1007/s44197-023-00090-8

**Published:** 2023-02-16

**Authors:** Nihar Ranjan Dash, Hiba Jawdat Barqawi, Anas A. Obaideen, Hanae Qousae Al Chame, Kamel A. Samara, Rama Qadri, Salma Eldesouki

**Affiliations:** 1 Clinical Sciences Department, College of Medicine, University of Sharjah, 27272 Sharjah, United Arab Emirates; 2 College of Medicine, University of Sharjah, Sharjah, United Arab Emirates

**Keywords:** COVID-19, Breakthrough infections, Covid vaccine, UAE, Sinopharm BBIBP-CorV, Pfizer-BioNTech

## Abstract

**Background:**

Despite significant efforts to contain the Coronavirus Disease 2019 (COVID-19) pandemic through mass vaccination, numerous nations throughout the world have recorded breakout infections. The incidence and severity of COVID-19 breakthrough infections in the United Arab Emirates (UAE) remain unknown despite extensive COVID-19 vaccine coverage. The goal of this research is to establish the characteristics of COVID-19 breakthrough infections in the UAE’s vaccinated population.

**Methods:**

Between February and March 2022, we conducted a descriptive cross-sectional study in the UAE with 1533 participants to examine the characteristics of COVID-19 breakthrough infection among the vaccinated population.

**Results:**

The vaccination coverage was 97.97%, and the COVID-19 breakthrough infection rate was 32.1%, requiring hospitalization in 7.7% of cases. The bulk of the 492 COVID-19 breakthrough infections reported was among young adults (67%), with the majority experiencing mild to moderate symptoms (70.7%) or remaining asymptomatic (21.5%).

**Conclusions:**

COVID-19 breakthrough infection were reported in younger age, male sex, non-healthcare professions, vaccination with inactivated whole virus vaccine (Sinopharm), and not receiving a booster dose. Information on breakthrough infection in the UAE might influence public health decisions and motivate measures such as providing additional booster doses of the vaccines to the people.

## Introduction

Since the start of the coronavirus disease 2019 (COVID-19) pandemic, dedicated efforts have been put towards developing vaccines that can protect the human host, slow down the spread of the virus and end its worldwide burden. In December 2020, the United Arab Emirates (UAE) approved the use of BBIBP-CorV (Sinopharm) inactivated whole COVID-19 virus vaccine after its proven efficacy of 79% in Phase III clinical trials conducted in the country in July 2020 [1]. The effectiveness of the vaccine in preventing hospital admissions and death following infection was then found to be up to 80% and 97% respectively, as demonstrated in a large-scale retrospective study conducted in Abu Dhabi, UAE [2]. In May 2021, the Pfizer-BioNTech messenger RNA vaccine was approved for use in the UAE, based on the Food and Drug Authority’s (FDA) announced efficacy of 95% [3]. Alongside Sinopharm and Pfizer BioNTech, three more COVID-19 vaccines Moderna, Oxford/AstraZeneca, and Sputnik were also distributed among the UAE population promptly, reaching around 97.97% of fully vaccinated individuals in May 2022 [4]. COVID-19 vaccine booster doses, specifically Pfizer BioNTech and Sinopharm, were advised 6 months after the original two doses to complete the vaccination regimen and be declared fully immunized.

With this high coverage of vaccination in the UAE population which is around 10 million, the rate of hospitalization, deaths, and severity in infection has significantly dropped from a peak of 3167 diagnosed cases and 13 deaths on the 14th February 2021 to 321 cases with no deaths by the 23rd May 2022 [4]. As anticipated, while the COVID-19 vaccine was highly effective in reducing coronavirus disease, deaths, infectivity, severity, asymptomatic infections and hospital admissions [5–8], vaccine breakthrough infection started to be reported worldwide [9, 10]. A COVID-19 breakthrough infection is defined as a positive test for COVID-19 in a person at least two weeks after becoming fully vaccinated [10, 11]. A person is fully vaccinated 2 weeks after receiving all recommended doses in the primary series of their COVID-19 vaccination and a person is considered up to date with their COVID-19 vaccination if they have received all recommended doses in the primary series and one booster when eligible [12].

More than 2.8 million breakthrough infections had been reported to the Centers for Disease Control and Prevention (CDC) in the United States as of December 2021 [7]. Breakthrough cases occur because no vaccine is 100% effective and in COVID-19 the variants such as the delta and omicron were the common etiology [13–15]. Breakthrough infections remains a threat to the COVID-19 pandemic. Vaccinated persons who get breakthrough infections may be asymptomatic or have minor symptoms, but they can still spread the virus to the rest of the community. In addition, people do not report the majority of breakthrough cases, thus the total number of reported breakthrough cases likely represents an undercount.

Breakthrough infections, despite their small numbers, can be misinterpreted as a failure of vaccine protection in some situations; yet, in reality they actually indicate that the vaccine is working properly. Furthermore, the incidence of breakthrough infections could be on the rise due to the decline in neutralizing antibody level in the host [16].

The incidence and severity of COVID-19 breakthrough infections in the UAE remain understudied, despite the widespread vaccination of the population. We believe such data is essential to describe these infections and inform public health decisions including delivering additional vaccine doses, adjusting vaccination tactics, continuing infection control methods, and managing the COVID-19 pandemic successfully.

## Materials and Methods

The study was performed after obtaining the necessary institutional ethics approval (REC-22-02-01). A descriptive cross-sectional study design was used in conjunction with a convenience sample strategy for all vaccinated individuals living in the UAE from February to March 2022.

A questionnaire consisting of 18 questions, divided into 3 different sections: demographics, COVID-19 vaccination, and COVID-19 breakthrough infection was administered through email and social media platforms such as WhatsApp group chats, Instagram and Twitter. The questionnaire was bilingual in English and Arabic and the questions were close-ended, in the form of single-choice and multiple-choice answers (appendix 1). A participant information sheet was presented before starting the questionnaire, and the agreement to fill out the questionnaire indicated the consent of the participants to join the study. Data was processed in python-3 using the Matplotlib-v3.3.4, pandas-v1.2.4, and statsmodels-v0.12.2 packages for analysis and interpretation. Given the categorical nature of all the variables, multivariate analyses were used to identify significant predictors. A *p* value of less than 0.05 was considered significant.

## Results

### Demographics

A total of 1544 individuals participated in the study of which 1533 responses were included in the analysis. Among the 1533 respondents, 863/1533 (56.3%) were males and 184/1533 (12%) reported to have comorbidities. As for the vaccination rates, 488/1533 (31.8%) of the respondents received 2 doses of the Pfizer vaccine, while 327/1533 (21.3%) received 2 doses of the Sinopharm vaccine followed by 2 booster doses of the Pfizer vaccine. Overall, 776/1533 (50.6%) of the sample were infected with COVID-19 at least once (Table 1).

**Table 1:** Demographics of the participants, along with the COVID-19 breakthrough rates

Demographics			Breakthrough (% of category)
	Categories	%	
Age	Younger than 18 years	498/1533 (32.49%)	140/498 (28.11%)
	18–25 years	617/1533 (40.25%)	189/617 (30.63%)
	26–30 years	98/1533 (6.39%)	25/98 (25.51%)
	31–40 years	139/1533 (9.07%)	68/139 (48.92%)
	41–50 years	105/1533 (6.85%)	43/105 (40.95%)
	51–65 years	60/1533 (3.91%)	22/60 (36.67%)
	Older than 65 years	16/1533 (1.04%)	5/16 (31.25%)
Sex	Male	863/1533 (56.29%)	269/863 (31.17%)
	Female	670/1533 (43.71%)	223/670 (33.28%)
Occupation	Healthcare sectors	121/1533 (7.89%)	42/121 (34.71%)
	Student	923/1533 (60.21%)	269/923 (29.14%)
	Housewife	88/1533 (5.74%)	33/88 (37.50%)
	Unemployed	65/1533 (4.24%)	14/65 (21.54%)
	Others	336/1533 (21.92%)	134/336 (39.88%)
Comorbidities	No	1351/1533 (88.13%)	431/1351 (31.90%)
	Yes	182/1533 (11.87%)	61/182 (33.52%)
Partially vaccinated	1 dose Pfizer vaccine	14/1533 (0.91%)	N/A
	1 dose Sinopharm vaccine	5/1533 (0.33%)	N/A
Fully vaccinated	2 doses Pfizer vaccine	488/1533 (31.83%)	152/488 (31.15%)
	2 doses Sinopharm vaccine	227/1533 (14.81%)	84/227 (37%)
Up to date vaccinated	2 doses Pfizer vaccine and 1 Pfizer booster	143/1533 (9.32%)	42/143 (29.37%)
	2 doses Sinopharm vaccine and 1 Sinopharm booster	169/1533 (11.02%)	77/169 (45.56%)
	2 doses Sinopharm vaccine and 1 Pfizer booster	77/1533 (5.02%)	28/77 (36.36%)
	2 doses Sinopharm vaccine and 2 Pfizer booster	327/1533 (21.33%)	109/327 (33.33%)
Unvaccinated	No vaccine	66/1533 (4.31%)	N/A
Infection rate	Never	756/1533 (49.32%)	N/A
	Once	611/1533 (39.86%)	359/611 (58.76%)
	Twice	126/1533 (8.22%)	104/1,533 (82.54%)
	More than 2 times	40/1533 (2.61%)	29/40 (72.50%)

### COVID-19 Breakthrough Infection & Hospitalization

**Fig. 1:**
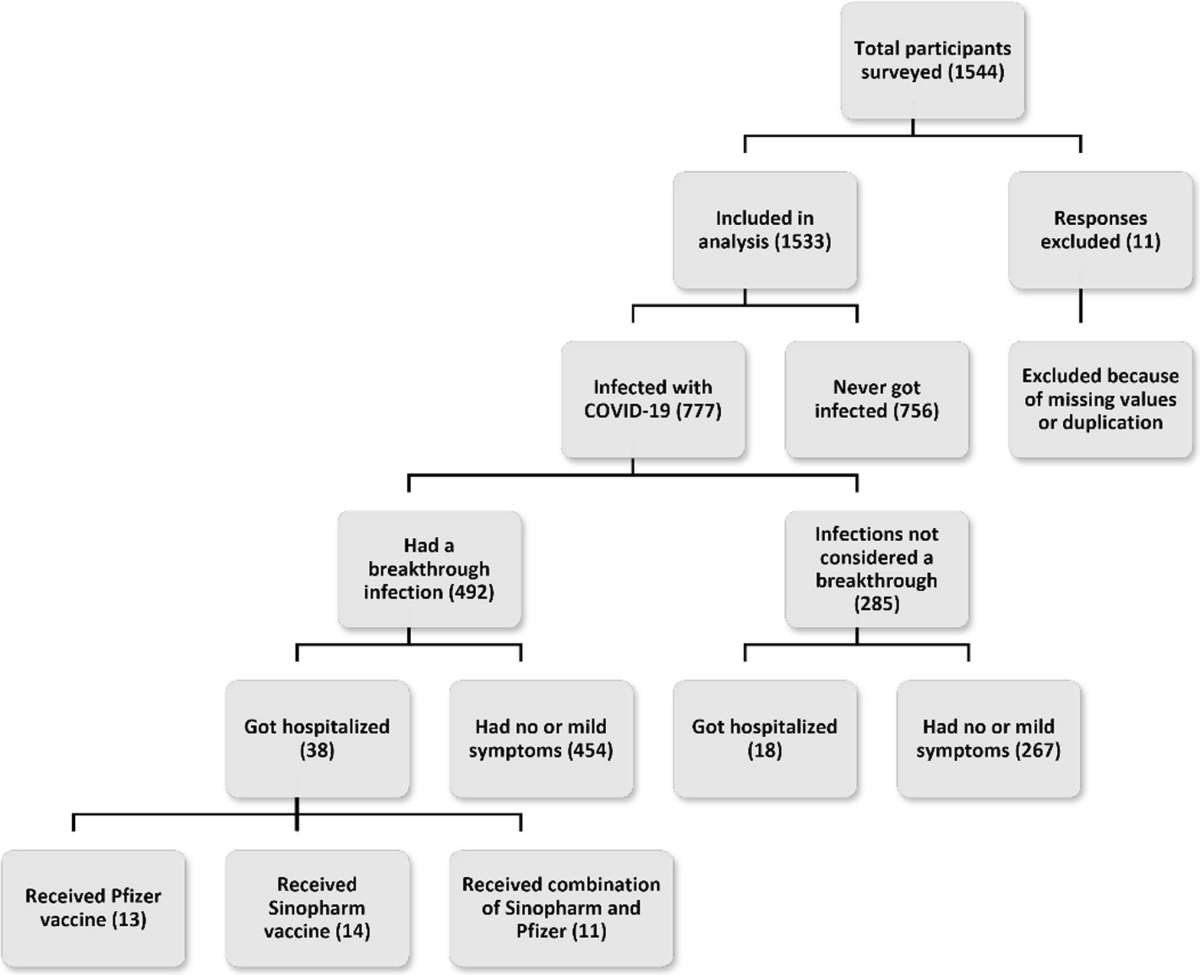
COVID-19 breakthrough infections and hospitalization

Of the 1533 participants, 492 (32.1%) reported having a breakthrough COVID-19 infection, of which 38 (7.7%) required hospitalization. Figure 1 shows how the participants were divided according to infection and hospitalization rates. All age groups had a breakthrough infection rate of over 25%, with the age groups 31–40 years and 41–50 years reporting the highest infection rates of 48.9% and 40.9%, respectively. The COVID-19 breakthrough infection rates among healthcare workers were 34.7% (42/121) and people with comorbidities such as diabetes, asthma, immunodeficiency and other chronic disorders was 33.5% (61/182).

**Fig. 2:**
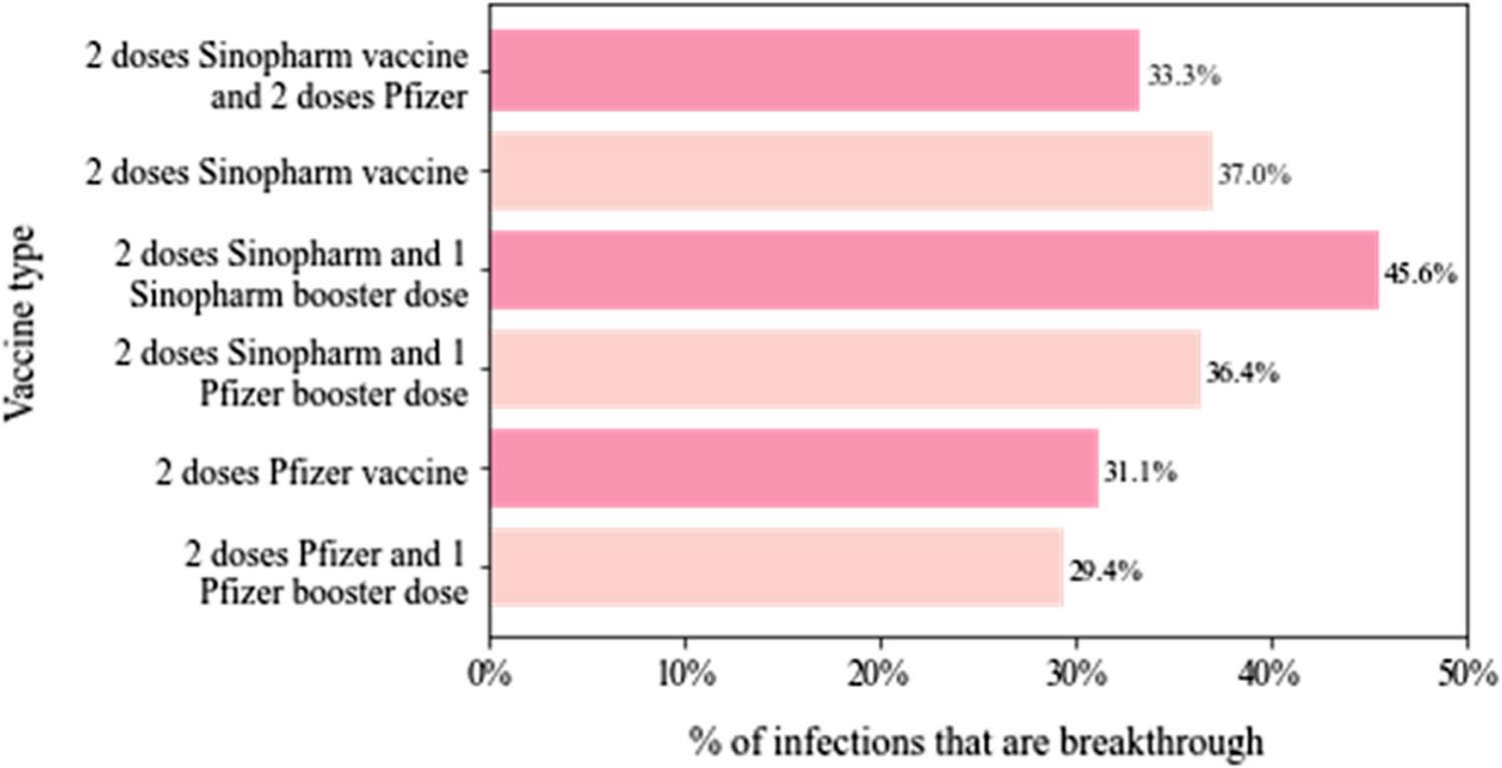
COVID-19 breakthrough infection rates in people with different combinations of vaccines

Surprisingly, individuals who received three doses of Sinopharm had the highest breakthrough rate (45.6%, 77/169), with roughly one out of every two infections, becoming a breakthrough infection (Fig. 2). When compared to people who were fully vaccinated (completed primary series) or up to date (primary + booster) with the Pfizer vaccine, the rate of COVID-19 breakthrough infections among people who received Sinopharm vaccine only or two doses of Sinopharm (primary doses) and a booster with Pfizer remains relatively high, at 37% (84/227) and 36.4% (28/77), respectively (Table 1 and Fig. 2).

Of the 492 COVID-19 breakthrough infections reported, the majority were seen among young adults (67%, 329/492), male sex (55%, 269/492) and most of these cases either experienced mild to moderate symptoms (70.7%, 348/492) or remained asymptomatic (21.5%, 106/492). Mild to moderate symptoms included fever, cough, sore throat, loss of taste, smell and other constitutional symptoms such as diarrhea, fatigue and body aches, which did not require hospitalization. Despite the subtlety of the clinical presentation, virus transmission to close contacts remains high (74.6%, 367/492) (Table 2).

**Table 2:** Characteristics of COVID-19 breakthrough infections

Breakthrough infection (*n* = 492)		
Age	< 25 years	329/492 (66.9%)
	25–50 years	136/492 (27.6%)
	> 50 years	27/492 (5.5%)
Sex	Male	269/492 (54.7%)
	Female	223/492 (45.3%)
Symptoms	Asymptomatic	106/492 (21.5%)
	Mild to moderate symptoms*	348/492 (70.7%)
	Require hospitalization	38/492 (7.7%)
Transmission to close contact	Yes	367/492 (74.6%)
	No	125/492 (25.4%)

*Fever, cough, sore throat, loss of taste, smell and other constitutional symptoms such as diarrhea, fatigue and body aches that did not require hospitalization

## Discussion

COVID-19 breakthrough infections pose a significant concern worldwide, despite the widespread efforts at eradicating infection through vaccination. In this study, among the 1533 individuals who participated, 492 reported COVID-19 breakthrough infection. Such infections are attributed to a number of factors, ranging from the emerging COVID-19 variants to insufficient immune response to vaccination. Prior to the emergence of the Delta variant, in April and May of 2021, breakthrough cases numbered around 48,000 in the US [7]. In comparison, over 1.8 million breakthrough infections were reported during the predominance of the variant between July and November 2021. This rise in breakthrough cases may be due to the reduced effectiveness of vaccines against the Delta variant, where an effectiveness of 88% against the variant was reported with the Pfizer-BioNTech vaccine compared to an effectiveness of 93.7% against the Alpha variant [17]. In addition, the high transmissibility, high viral load, and shorter incubation period of the Delta variant may have contributed to the elevation in breakthrough cases [18].

Similarly, in the month of December 2021, as the Omicron variant was beginning to emerge worldwide, more than 900,000 breakthrough cases were reported in the US [7]. This is parallel to the rise in the number of cases in the UAE during the period of Omicron predominance, peaking at 3020 in mid-January, a sharp increase from under 100 cases the month before [19]. Surprisingly, the upsurge occurred during a period where 93.5% of the UAE’s population was fully vaccinated, where the majority of cases were likely to be breakthrough [20]. The main vaccines distributed among the UAE citizens are the Sinopharm and Pfizer-BioNTech vaccines, whose effectiveness against the Omicron variant was shown to decline. Neutralizing antibodies against the Omicron variant were only detected in up to 24% of individuals who received the primary 2 doses of the Pfizer-BioNTech vaccine in a trial study, and in another study, vaccine effectiveness dropped from 65.5% at 2 to 4 weeks to 8% at 25 weeks or more after the two doses [21, 22]. Similar findings were seen in Sinopharm-vaccinated individuals, where the levels of neutralizing antibodies against Omicron were reduced by 53% almost 26 weeks after the administration of a third shot following the primary two-dose regimen [23]. To ameliorate the burden of breakthrough infections, an mRNA-based booster vaccine was shown to remarkably raise the levels of antibodies and B and T cell responses against the variant, following administration of the primary doses of either Pfizer-BioNTech and Sinopharm BIBP [24].

In this study, 45.6% (77/169) of individuals who received two doses of Sinopharm BIBP followed by a Sinopharm CNBG booster vaccine developed a breakthrough infection, making up the greatest proportion of breakthroughs among all the vaccine regimens. While those who received two doses of Pfizer-BioNTech followed by a booster with the same Pfizer-BioNTech vaccine reported a relatively low breakthrough infection rate of 29.4% (42/143). In-between, were the heterologous vaccination, two doses of Sinophram and a Pfizer-BioNTech booster and two doses of Sinophram and two doses of Pfizer-BioNTech booster, reported breakthrough rates of 36.4% and 33.3%, respectively. Studies have indicated that mRNA vaccines, such as those made by Pfizer and Moderna, provide far better protection than those made by AstraZeneca and Janssen, which utilize a more standard viral vector formula [25] and in our case an inactivated viral vaccine such as Sinopharm BIBP. Combination of COVID-19 vaccination practice such as primary dosing with an inactivated vaccine followed by a booster dose with an mRNA vaccine has potentially increased protection against SARS-CoV-2 variants such as Omicron by augmenting the levels of specific antibodies and B and T cell responses [24] but has not eliminated the occurrence of breakthrough infections.

There has been a noticeable surge in hospital admissions due to COVID-19 infections worldwide. A rate of 5.8% of all patients with a confirmed COVID-19 infection in the United States were hospitalized [26]. In contrast, hospital admissions following a breakthrough COVID-19 infection fell dramatically below 1% as reported by the New York State Department of Health [27]. However, in this study, 38 of the 492 COVID-19 breakthrough infection cases (7.7%) required hospitalization, which is towards the higher side. Advanced age and having comorbidities were the two common factors consistently associated with patients requiring hospitalization. This is in line with a reported observation that the vast majority of the hospitalizations were in individuals aged 65 and above (69%) and having comorbidities [28]. Interestingly, in this study, the rates of breakthrough infections among individuals suffering from comorbidities and individuals without were comparable. As a result, we believe the etiology of breakthrough infections in our study is linked to the developing Omicron variant and its associated immune evasion, rather than the loss in vaccine effectiveness due to comorbidities.

In the multivariate analysis, we found that breakthrough infections were more common in younger age groups, such as 67% (25 years old) and 28% (25–50 years old), compared to 5.5% in older age groups (> 50 years old), which we attribute to young people’s social behavior, higher social contacts, and work-related contacts, which is consistent with other cohorts’ reported data [29–31]. Similarly, reinfections rates were higher than a single episode of breakthrough infection especially during the wave of the Omicron variant peak [32]. Another interesting observation was, despite being exposed to COVID-19 patients on a daily basis, healthcare workers were found to have a decreased chance of developing a breakthrough infection when compared to a non-healthcare worker. This could indicate that healthcare workers were better protected with higher vaccine coverage and practiced infection control measures especially at work [29, 33].

In this study, despite the fact that the majority of patients with a breakthrough infection were asymptomatic or had mild to moderate symptoms, they were able to spread the infection to close relatives and friends (74.6%). This is concerning because breakthrough infections have contributed to increased case numbers and pandemic extension at a time when vaccine effectiveness is diminishing, lockdowns, infection control measures, and social restrictions are being lifted.

The small cohort size of our study, despite vaccine coverage of nearly 97% of the UAE population, is one of the study’s potential drawbacks. The lack of antibody titer specifically the anti-S level data and its link with breakthrough infections is another limitation. It is evident that post-COVID-19 vaccination antibody titer wanes off with time and has been directly correlated with the occurrence of breakthrough infections [34]. We do have a plan to address both these limitations in a future study linking the antibody titer measurements with breakthrough infections in a bigger cohort size.

In conclusion, this study found that younger age, male sex, non-healthcare professionals, vaccination with inactivated whole virus vaccine (Sinopharm), and not obtaining a booster dose were all linked with an elevated risk of COVID-19 breakthrough infection.

## Data Availability

Data will be available from the corresponding author on reasonable request.

## Appendix 1

English version

## COVID-19 Breakthrough Infections in Vaccinated Population

We are a group of medical students at the University of Sharjah conducting a research project about the COVID-19 breakthrough infections in vaccinated populations.

You have been randomly selected to participate in this study and your participation is strictly voluntary. If you agree to participate, you will be asked to fill out a questionnaire that will take no more than 4–7 min of your time.

There are no risks or benefits associated with participation in this study. Your identity will remain unknown, and we assure you that your responses will be confidential and will be usedonly for research purposes.

You have the right to withdraw from the study at any time during filling out the questionnaire,but you cannot withdraw after submitting it since it will be unidentifiable.

Please note that no incentives will be given for the completion of this survey.

If you have any questions regarding this study or would like to be informed about its results,please feel free to contact our research supervisors Dr. Hiba Barqawi at hbarqawi@sharjah.ac.ae or Dr. Nihar Dash at ndash@sharjah.ac.ae.

For any ethical concerns, you may contact the Research Ethics Committee at the University ofSharjah—rec@sharjah.ac.ae.

Filling out this questionnaire indicates your agreement to participate in this study.

اﻹﺟﺎﺑﺔ اﻹﺳﺗﺑﯾﺎن ﺑﺎﻟﻠﻐﺔ اﻟﻌرﺑﯾﺔ اﺿﻐط ھن

https://forms.gle/YSX4zkX9PyS7f85A9

*Required.

COVID-19 Vaccination.

## Abbreviations

COVID-19: Coronavirus Disease 2019
UAE: United Arab Emirates
